# Limiting opportunities for cheating stabilizes virulence in insect parasitic nematodes

**DOI:** 10.1111/eva.12348

**Published:** 2016-01-27

**Authors:** David Shapiro‐Ilan, Ben Raymond

**Affiliations:** ^1^ Southeastern Fruit and Tree Nut Research LaboratoryUSDA‐ARSByronGAUSA; ^2^ Royal Holloway University of LondonEghamSurreyUK; ^3^ Imperial College LondonSilwood Park CampusAscotBerksUK

**Keywords:** biological control, cooperation, evolution of virulence, *Heterorhabditis floridensis*, pest management, stability

## Abstract

Cooperative secretion of virulence factors by pathogens can lead to social conflict when cheating mutants exploit collective secretion, but do not contribute to it. If cheats outcompete cooperators within hosts, this can cause loss of virulence. Insect parasitic nematodes are important biocontrol tools that secrete a range of significant virulence factors. Critically, effective nematodes are hard to maintain without live passage, which can lead to virulence attenuation. Using experimental evolution, we tested whether social cheating might explain unstable virulence in the nematode *Heterorhabditis floridensis* by manipulating relatedness via multiplicity of infection (MOI), and the scale of competition. Passage at high MOI, which should reduce relatedness, led to loss of fitness: virulence and reproductive rate declined together and all eight independent lines suffered premature extinction. As theory predicts, relatedness treatments had more impact under stronger global competition. In contrast, low MOI passage led to more stable virulence and increased reproduction. Moreover, low MOI lineages showed a trade‐off between virulence and reproduction, particularly for lines under stronger between‐host competition. Overall, this study indicates that evolution of virulence theory is valuable for the culture of biocontrol agents: effective nematodes can be improved and maintained if passage methods mitigate possible social conflicts.

## Introduction

Kin selection theory resolves the conflict over the maintenance of behaviors that are beneficial for groups of organisms, but costly for individuals, by reasoning that costly cooperation is stable if it is primarily directed at individuals that share the same alleles for cooperation, that is under high relatedness (Hamilton 1964a,b). Social conflicts have repeatedly been identified in microbial pathogens, which often secrete virulence factors that are analogous to ‘public goods’; these goods are costly to individuals, beneficial to groups and can be exploited by cheating mutants that benefit from collective action, but do not contribute to it (Köhler et al. 2009; Raymond et al. 2012; Diard et al. 2014a). Critically, if relatedness is low, cheating mutants can arise and locally outcompete cooperators within hosts (Köhler et al. 2009; Raymond et al. 2012; Diard et al. 2014a).

An additional ecological factor that is important for the maintenance of cooperation is the scale of competition or the population structure of individuals engaged in cooperation. Limited dispersal or population viscosity can lead to high relatedness but can also mean that cooperators primarily compete with their relatives, which can undermine the benefits of altruism (Taylor 1992; Wilson et al. 1992; Frank 1998). In contrast if competition is primarily global, for example between patches or groups of unrelated organisms, then altruism can be favored (Frank 1998). While this theory is not experimentally tractable in vertebrates and higher organisms, experimental evolution of the cooperative secretion of iron‐scavenging siderophores suggests that both relatedness and the scale of competition are important for microbial cooperation in simple systems (Griffin et al. 2004; Kümmerli et al. 2009).

Social evolution theory has had a dramatic impact on microbiology and microbial ecology, with relevance to the evolution of virulence (Griffin et al. 2004; Sandoz et al. 2007); dose–response (Cornforth et al. 2015); the evolution of resistance to antibiotics (Dugatkin et al. 2005; Diard et al. 2014b) as well as having clinical implications (Köhler et al. 2010). Moreover, since cooperation is important for the virulence of specialist invertebrate pathogens (Raymond and Bonsall 2013; Zhou et al. 2014), and of opportunistic pathogens in model insects (Harrison et al. 2006; Racey et al. 2009; Pollitt et al. 2014), social biology could be pertinent for the maintenance and improvement of biocontrol agents (Garbutt et al. 2011). Entomopathogenic nematodes (EPN) are ideal candidates here. EPN are important biological control tools with particular value for invertebrate pests in moist or hard‐to‐treat habitats such as soil (Ehlers 2003). Prior studies on infection dynamics have primarily focused on pre‐infection behavior (Bohan and Hominick 1997; Glazer 1997; Griffin 2012), yet our focus is on the fitness of nematodes within the host during and after infection.

EPN depend partly on symbiotic bacteria to infect and kill hosts; both bacteria and nematodes secrete diverse virulence factors (Lewis and Clark 2012). Nematodes, for example, secrete a range of enzymes in their parasitic phase, the most abundant of which are diverse proteases with putative roles in suppression of host immunity and tissue invasion (Hao et al. 2009). Notably, ‘deteriorated’ or attenuated nematodes, which have reduced ability to infect hosts, have shown reduced expression of secreted proteases (Simões et al. 2000; Adhikari et al. 2009). Symbiotic bacteria secrete numerous toxins, lipopolysaccharides, degradative enzymes and small molecules which are important for host invasion, immune suppression and evasion, nematode nutrient supply and antagonistic competition with other bacteria (Forst et al. 1997; Ffrench‐Constant and Bowen 2000; Daborn et al. 2002; Eleftherianos et al. 2007). The contribution of both nematode and bacteria is considerable, but their relative importance in infection varies with both EPN and host species (Eleftherianos et al. 2010).

Critically, effective EPN strains are hard to maintain without serial propagation *in vivo* (passage). EPN can only be stored for weeks or months at temperatures above freezing, while freezing in liquid nitrogen and the subsequent defrosting has been known to lead to loss of fitness (Bai et al. 2004). The main drawback of serial propagation of nematodes is attenuation, that is the reduction in virulence over repeated rounds of infection (Wang and Grewal 2002; Bai et al. 2005; Bilgrami et al. 2006). Given the secreted nature of many bacterial and EPN virulence traits, we hypothesized that social conflict and the invasion of ‘cheaters’ with low investment in virulence, might be the cause of the unstable virulence of EPNs. We further hypothesized that high relatedness (maintained by low MOI passage) and global competition between infected cadavers should maintain cooperation and consequently high levels of virulence and within‐host reproduction: we tested this hypothesis with an experimental evolution approach.

## Materials and methods

**Figure 1:**
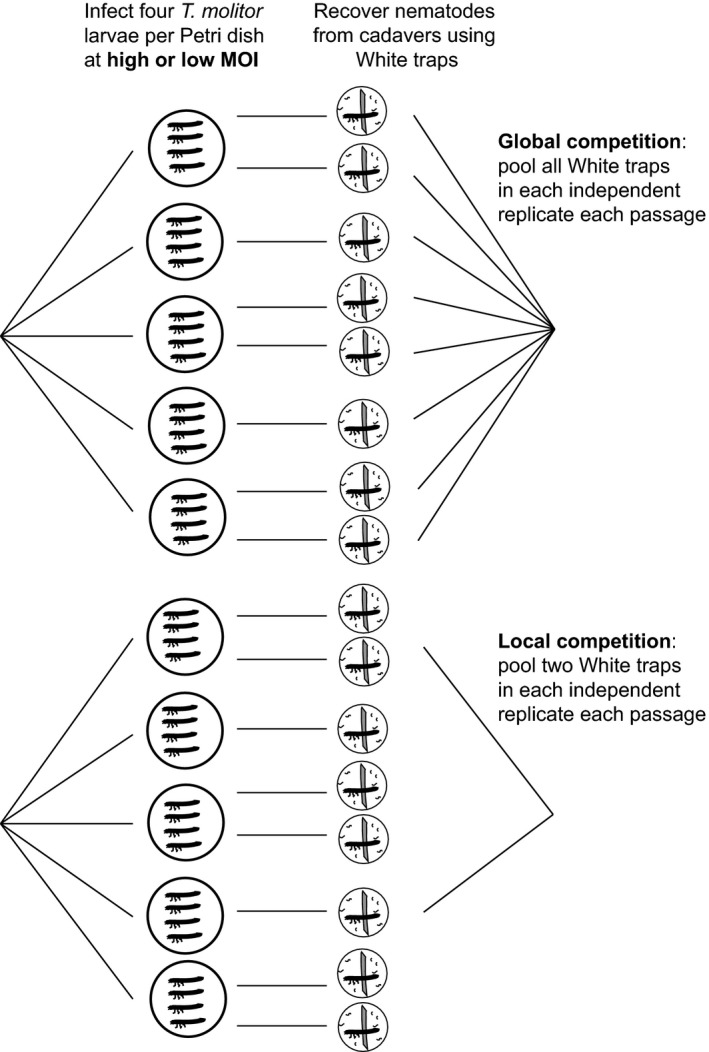
The design of the selection experiment, illustrating the global and local competition treatments. This scale of competition treatment was crossed with a multiplicity of infection (MOI) treatment (two levels), in which we imposed low relatedness by using 5000 infective juveniles (IJs) per dish or higher relatedness by using 250 IJs per dish. We used four independent selected lines in each treatment. Note that harvest (White) traps for individual cadavers in the local competition treatment were briefly screened for effective IJ production before being included in inocula pools for the next round of infection.

In order to test whether or not cooperation was likely to influence the maintenance of virulence in EPN, we attempted to independently manipulate both relatedness and the degree of global competition in a factorial selection experiment with 16 independent lineages, using an experimental design based on Griffin et al.'s (2004) classic study of siderophore production. We used a recently isolated field population of *Heterorhabditis floridensis* Nguyen et al. (Shapiro‐Ilan et al. 2014). The isolate was obtained from soil during a survey conducted in September 2011; using the *Galleria mellonella* baiting method (Shapiro‐Ilan et al. 2003), the nematodes were extracted from a composite soil sample (five replicates) taken from a hay field in Lizella, Georgia USA. Experimental evolution was conducted by passaging EPN in larvae of the mealworm, *Tenebrio molitor* L. (9–10th instar with mass 70–90 mg each). Recently collected EPN strains tend to have substantial genetic variation (Glazer et al. 1991; Shapiro et al. 1997) and so infections with large numbers of parasites should facilitate within‐host competition between genotypes with differing virulence phenotypes. We therefore manipulated relatedness by manipulating nematode dose or ‘multiplicity of infection’ (MOI) at each passage. A high MOI is known to decrease relatedness and increase the potential for social conflict as it facilitates competition and cheating between genetically diverse parasites within hosts (Turner and Chao 1999; Brown et al. 2002). We confirmed that our experimental doses led to different invasion rates via dissection: doses of 250 infective juveniles (IJs) led to an average of 7.6 invading IJs per host (SE 1.94), while higher doses (5000 IJs) led to an average of 100.3 invaders per host (SE 4.38). We varied the degree of global competition by altering the number of EPN‐killed cadavers pooled at each passage, reasoning that pooling pathogens from a larger number of hosts should increase global competition, at least in terms of reproduction after death. The design of the passage experiment is outlined in Fig. 1.

Nematodes were assayed for virulence and reproductive ability in hosts after 6 and 12 host passages. IJ counting was conducted under a stereomicroscope according to the established techniques (Kaya and Stock 1997). Virulence bioassays used two doses (100 and 500 IJ per insect) in 60‐mm Petri dishes with 0.35 mL of nematode suspension. The assays used 10 insects per dose and were repeated twice. Thus, for the full bioassay of 16 lines in passage 6, we scored mortality over 4 days for *N *=* *640 insects in total. Parametric survival analysis (using a log‐logistic distribution) was used to examine time to death; random effects of selection lineage were accounted for using the *cluster* function; model fits were checked by inspection of AIC and custom qqplots (Tableman and Kim, 2004) and significance testing carried out by model simplification and chi‐square tests on changes in deviance. Data on nematode reproduction (four cadavers per lineage per assay) were analyzed with maximum‐likelihood mixed model anova, using selection replicate as a random effect. All data were analyzed in R 3.0.2 (http://www.R-project.org).

## Results

### Evolution of virulence

**Figure 2:**
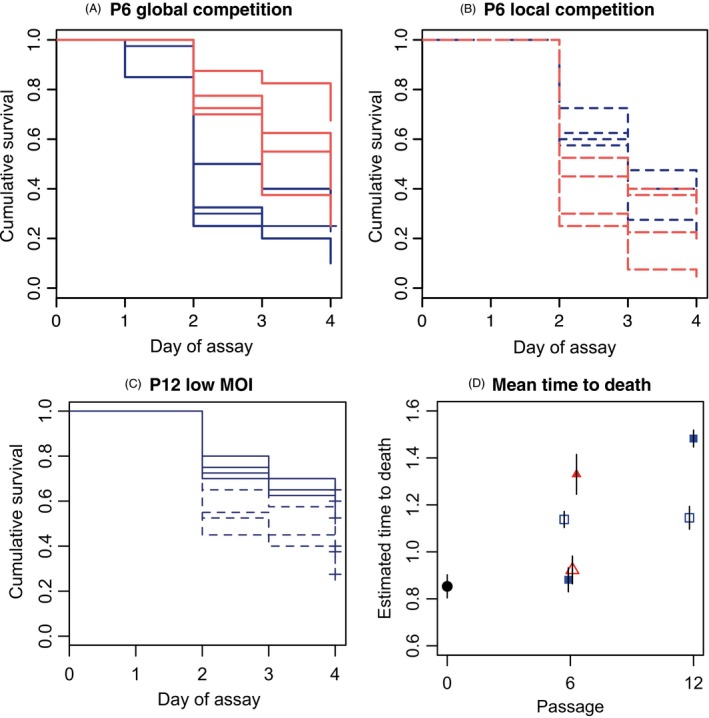
Variation in virulence over the course of the selection experiment. (A) Kaplan–Meier survivorship curves for passage 6 bioassays conducted with the eight lineages in the global competition treatment. (B) Kaplan–Meier survivorship curves for passage 6 bioassays conducted with the eight lineages in the local competition treatment. (C) Survivorship curves for the surviving low MOI lineages in passage 12. (D) Parameter estimates of time to death (±SE) in the baseline population (filled circle at P0) and all subsequent selection treatments. Red lines/red triangles represent the high MOI lineages, dark blue lines/squares high relatedness (low MOI) lineages, dashed lines and open symbols represent local competition and solid lines and filled symbols represent global competition.

Multiplicity of infection had an important impact on the evolution of changes in virulence and EPN reproduction after six rounds of passage. After accounting for bioassay dose (df = 1, *χ* ^2^ = 88.3, *P *≪ 0.0001), we found that virulence, in terms of mean time of death, was higher in low MOI treatments overall (df = 1, *χ* ^2^ = 7.84, *P *=* *0.0051). However, this effect of MOI varied between our global and local competition treatments (interaction df = 1, *χ* ^2^ = 84.3, *P *≪ 0.0001), there being a much clearer effect of MOI in the high global competition lineages (Fig. 2A,B).

In addition to the phenotypic differences seen at passage 6, there was a substantial increase in extinction risk associated with low relatedness. During passage 7, none of the lineages in the global competition/high MOI treatment produced any viable IJs, while cadavers from the local competition/high MOI treatments produced too few nematodes to infect the next round of insects. The probability of all eight of the low MOI lineages going extinct, but none of the others, was highly significant (one‐tailed Fisher's exact test, df = 1, *P = *7.7 × 10^−5^). We continued with the low MOI lineages until passage 12 to test whether any differences would emerge between the global and local competition treatments. After 12 passages, significant phenotypic differences in time to death emerged between the local and global competition treatments, although not in the expected direction: the local competition treatment, which entailed competition from only two cadavers per replicate per passage (Fig. 1), had higher virulence than the global competition treatment (df = 1, *χ* ^2^ = 24.1, *P *≪ 0.0001; Fig. 2C).

While this experiment was designed to make quantitative comparisons between treatments for bioassays conducted simultaneously, we can at least make qualitative comparisons between assays conducted at different time points and with our initial baseline data. Comparisons with the baseline population suggest that virulence was attenuated in two of our selection treatments by passage 6, although virulence in the low MOI local competition lines was stable thereafter. Quantitative comparisons between virulence bioassays conducted at passage 6 and passage 12 (for the low MOI lineages) indicated that differences in virulence depended on the scale of competition treatments (passage * competition treatment interaction, df = 1, *χ* ^2^ = 49.6, *P *≪ 0.0001). Thus, while virulence was stable in the low MOI/local competition lineages (post hoc *z* test‐ estimate −0.033, SE = 0.053, *z *=* *−0.63, *P *=* *0.53, Fig. 2D), lineages in the global competition treatment did have reduced virulence in passage 12 relative to passage 6 (post hoc *z* test‐ estimate 0.51, SE = 0.032, *z *=* *15.0, *P *<<< 0.0001, Fig. 2D). Qualitatively similar patterns can be seen in terms of the differences in infectivity, that is the proportion of successfully invaded cadavers in bioassays (Supplementary Material, Figure S1).

### Reproductive ability and life‐history trade‐offs

**Figure 3:**
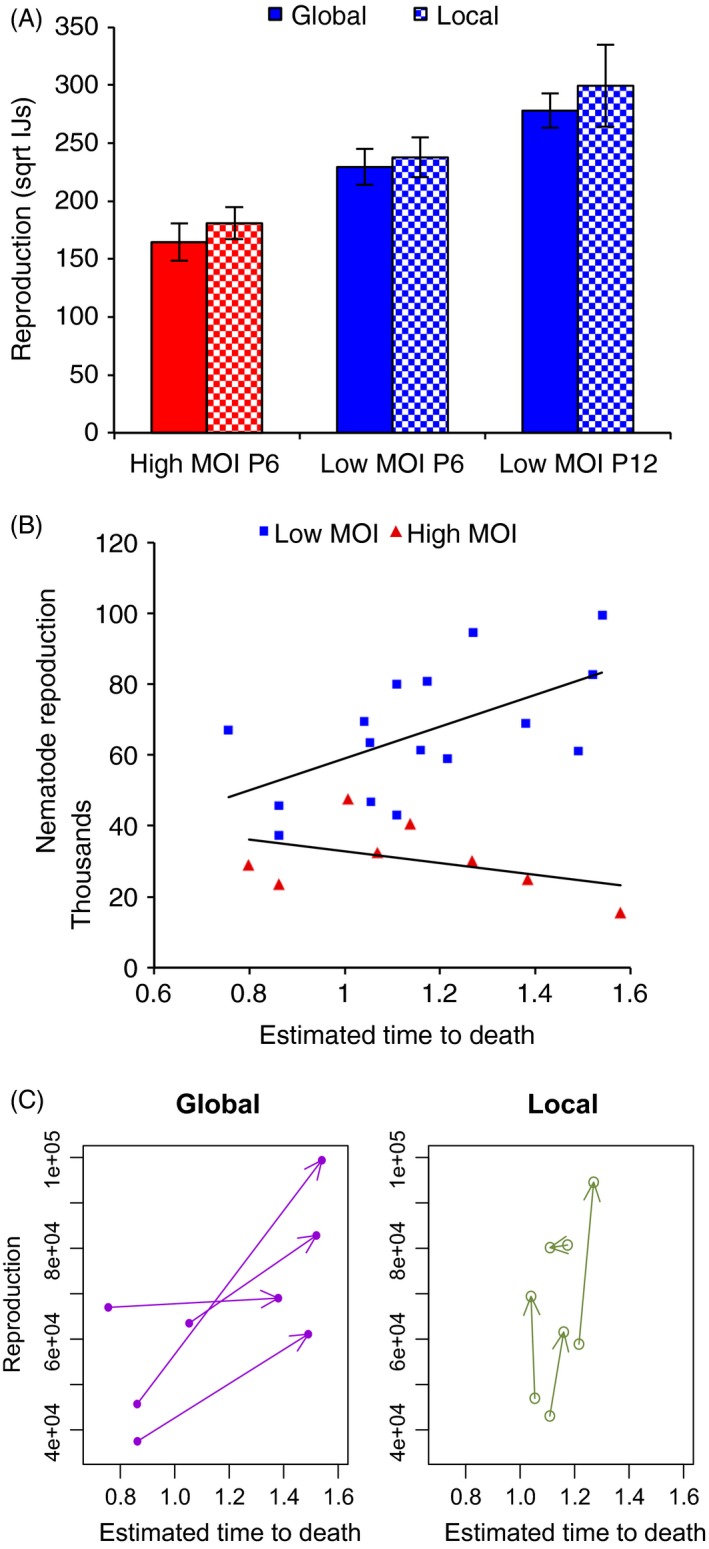
(A) *In vivo* reproduction (lineage means ± SE,* N = *4) of infective juveniles (IJs) produced from cadavers in after passage 6 and passage 12. Data are mean square root IJ count per host (±SE). (B) The relationship between *in vivo* reproduction and virulence for lineages assayed at passage 6 and passage 12. Reproduction data are untransformed IJ counts while time to death data are parameter estimates for each lineage calculated using log‐logistic survivorship models. Note that longer time to death indicates lower virulence. Red bars/red triangles represent the high MOI lineages, dark blue bars/squares high relatedness (low MOI) lineages, open bars represent local competition, and filled bars represent global competition. (C) The changing phenotypes of the low MOI lineages, the arrows lead from replicates assayed at passage 6 to the same lineage assayed at passage 12.

We expected that increased investment in public goods, maintained by high relatedness, might improve reproduction. In line with this prediction, in the passage 6 bioassays, low MOI lineages had increased reproductive rates (i.e. the production of IJs per cadaver) relative to the high MOI lineages (Fig. 3A; mixed model ANOVA‐ df* *= 1, likelihood ratio = 6.98, *P *=* *0.0082). We did not see any differences in total reproduction per host of EPN between the local and global competition treatments (mixed model ANOVA‐ df = 1, likelihood ratio = 0.30, *P *=* *0.58), a result that was consistent with our time to death data.

At passage 12, lineages with high virulence did not have improved reproduction *in vivo*, and there was no overall difference in nematode reproduction between the local and global competition treatments after 12 passages (mixed model ANOVA, df = 1, likelihood ratio = 0.4, *P *=* *0.53; Fig. 3A,C). We compared assays of reproduction and virulence conducted at passages 6 and 12 to test whether these traits were stable, unstable or responding positively to selection pressure in the laboratory. Reproduction rates of IJs in cadavers were significantly higher in passage 12 than in passage 6 (mixed model anova, df = 1, likelihood ratio = 4.92, *P *=* *0.027; Fig. 3A).

We looked for covariation or trade‐offs in the two key life‐history traits of virulence and reproduction in the data from all bioassays of these parasitic nematodes. These traits co‐varied in a manner that depended on the MOI during insect passage. For the high MOI strains, nematode reproduction decreased with declining virulence i.e. longer time to death, while in the low MOI strains increased reproductive rate appeared to trade‐off against virulence (Fig. 3B, MOI * time to death interaction *F*_1,20_ = 6.1, *P *=* *0.023). Comparisons between individual lineage at passage 6 only do not show clear evidence of a positive correlation between virulence and reproductive rate between individual lineages (Supplementary Material, Figure S2; *F*_1,14_ = 1.70; *P *=* *0.22), this trade‐off may have partly developed through evolutionary change between passage 6 and 12. Qualitative examination of the phenotype change in lineages between passages 6 and 12 suggests that in at least three of the four global competition lineages increased reproduction came at the cost of longer time to death (= lower virulence) (Fig. 3D).

## Discussion

### Cooperation and evolution of virulence and parasite reproduction

For pathogens, increased investment in secreted public goods may lead to improved infectivity, as toxins or quorum regulated enzymes may be required for effective host invasion (Raymond and Bonsall 2013; Zhou et al. 2014). Increased infectivity can lead to correlated changes in virulence measurable as faster time to death. However, a second role for microbial public goods is in the acquisition of essential host resources (MacLean and Gudelj 2006; West et al. 2007), such as the harvesting of growth‐limiting iron by bacterial siderophores (West and Buckling 2003). With these classical public goods, we expect that increased investment in cooperative exoproducts will lead to more effective exploitation of the finite resources inside a host and therefore larger parasite population sizes (West and Buckling 2003; Diggle et al. 2007; Sandoz et al. 2007). We therefore predicted that selection treatments favoring cooperation (high relatedness, high global competition) would lead to increased total EPN reproduction per host; this prediction held true for our passage 6 data (Fig. 3A) in which low MOI treatment had higher reproductive rates than the high MOI treatment. Furthermore, the declining reproduction in low MOI lineages was also associated with declining virulence. Since both declining virulence and reproduction is likely to decrease parasite fitness, an evolutionary trajectory in which these traits decline together appears to be highly maladaptive, an interpretation that is supported by the subsequent extinction of all eight of the high MOI lineages. Notably, our cheat invasion hypothesis can explain this loss of fitness quite well, although this is harder to explain with other evolution of virulence theories.

In contrast, when we passaged at low MOI, there was some evidence of a trade‐off between high reproductive rate and virulence. Across all lineages high virulence tended to be associated with intermediate levels of parasite fecundity. Insect parasites, as obligate killers, are not expected to follow the trade‐offs predicted by classical evolution of virulence theory (Ebert and Weisser 1997) and non‐linear relationships between virulence and reproduction are not unusual in entomopathogens (Hodgson et al. 2004; Raymond et al. 2012). If our bioassay data (at 500 and 100 IJs per dish) are indicative of what is happening during a low MOI passage (at 250 IJs per dish), then the global competition strains that have declined in infectivity by half (from 82% to 36%) have compensated with a near doubling in reproductive rate (from 5.0 × 10^4^ IJs to 8.2 × 10^4^ IJs, Fig. 3C). Conceivably, the global competition treatment, which pooled many cadavers at each passage, could facilitate increased selection for fecundity, which can lead to correlated changes in other nematode life‐history traits (Bashey and Lively 2009).

Increasing the opportunities for cheating with increased multiplicity of infection had dramatic consequences, as high MOI led to reduced virulence, reduced ability to exploit hosts and ultimately extinction (Figs 2 and 3), implying that high relatedness is fundamental to maintaining infectivity in this species. While there are few data on levels of relatedness in naturally infected hosts for any parasite (Balmer and Tanner 2011; Raymond et al. 2012), the infection rates experienced by many EPN in the laboratory context are likely to be much higher than in the field. In particular, the heterorhabditis are capable of clonal infections, since they reproduce by hermaphroditic selfing in the first generation in the host, while sexual (amphimictic) mating occurs only in subsequent generations (Lewis and Clark 2012). Selfing within the host has two important implications, firstly it can facilitate the evolution of cooperative virulence by lowering relatedness, but secondly a round of clonal competition will further reduce genetic diversity in the host relative to that in original invaders as different genetic lineages may vary in the timing of invasion and/or compete for resources (van Leeuwen et al. 2015). Theory also predicts that relatedness will have a more limited impact on cooperation when competition is local, as competition between relatives offsets the benefits of altruism (Taylor 1992; Wilson et al. 1992; Frank 1998). Consistent with this prediction, relatedness treatments interacted with the scale of competition: with local competition relatedness had a much weaker impact on virulence assessed at passage 6.

Pooling an increased number of cadavers in order to increase levels of global (between hosts) competition did not have the predicted effect on the evolution of nematode virulence. Contrary to expectation, lineages propagated from the fewest cadavers maintained highest virulence over the course of the whole study (Fig. 3C,D), although we did see the theoretically predicted interaction between the scale of competition and relatedness at passage 6. However, there were critical differences between this study and similar experiments conducted with bacteria in artificial media (Griffin et al. 2004). First, with any naturalistic parasite infection it is impossible to eliminate between‐host competition. Hosts are patches of resources, and, inevitably, there is competition in terms of the ability of parasites to establish infections at a between‐host scale, especially, as we saw here, when only 3–4% of the IJs exposed to an insect successfully invade the host. A second, additional constraint of live passage is that, at each round of infection, we need to propagate sufficient parasites to infect the next batch of hosts. In order to generate sufficient infectious material in the local competition treatment, we propagated nematodes from two cadavers, which were known to contain some nematodes. Again, this led to another form of global competition, in that infections had to produce a minimum number of infective juveniles. Third, in contrast to broth experiments with bacteria, independently manipulating relatedness and scale of competition was not straightforward: it was highly impractical to initiate infections with single clones, so that there was potential for social conflict at the lowest dose in our selection design. Finally, this study did not begin with controlled proportions of cheaters and cooperators, so any social conflict entirely depended on any uncharacterized genetic diversity at the start of the experiment or on spontaneous mutations arising during passage.

While we anticipated that we would have less power to manipulate the scale of competition compared to relatedness, in retrospect, bottlenecking parasite populations through fewer cadavers at each passage (the local competition treatment) most likely resulted in lower genetic diversity in each inoculum pool and thus higher relatedness (Strassmann and Queller 2011; van Leeuwen et al. 2015), and may have selected for increased reproduction at the cost of lower virulence. While we anticipated that it would be difficult to manipulate the scale of competition, this experiment suggests that between‐host competition in establishing infections, which we could not manipulate so readily, is probably more important for the evolution of virulence than between‐host competition in parasite fecundity, which we could manipulate to some degree.

### Implications for maintenance of parasitic nematodes

Comparing assays of reproduction and virulence conducted at passage 6 and 12 can indicate whether these traits were stable, unstable or responding positively to selection pressure in the laboratory. We found that the low MOI/local competition treatment produced the most stable virulence, although virulence may have declined slightly in the first six passages. Reproductive rate also responded positively to selection in the laboratory, as has been found in selection experiments on other nematode traits when EPN are passaged at relatively low MOI (Gaugler et al. 1989). A caveat here is that we did not assay evolved EPN strains against an ancestral genotype that has been revived from a frozen stock, as is the norm for experimental evolution of bacteria, since freezing in liquid nitrogen can alter nematode phenotypes (Wang and Grewal 2002). Thus, we cannot exclude the possibility that variation in the experimental set‐up between assays 6 months apart may have contributed to any observed differences. Nevertheless, comparisons between experiments separated in time are typically used to assess changes in nematode traits after artificial selection (Bai et al. 2005; Chaston et al. 2011), and so provide a useful benchmark.

Based on this study, we can provide clear advice to biological control practitioners and researchers wishing to preserve the fitness and virulence of recently isolated EPN: infecting insects with as few nematode genotypes as possible by using low doses and making inocula pools from as few cadavers as possible should stabilize virulence. Our results are consistent with previous work that has shown that removing genetic variation by inbreeding recently isolated nematodes can successfully stabilize virulence (Bai et al. 2005; Anbesse et al. 2013). However, the method used here (passage at low MOI) has several advantages over a pre‐emptive inbreeding strategy. First, it is possible to preserve genetic variation in a laboratory stock if lines from different cadaver pools are stored and passaged separately. Thus, if selection primarily proceeds by the variation in infection success and reproduction of different genotypes in different hosts, then it should be possible to preserve virulence as well as impose laboratory selection on other desirable traits, such as drought tolerance (Mukuka et al. 2010). Second, the creation of inbred lines opens the possibility of inbreeding depression, known to be a potentially serious problem for fecundity and trait stability in nematodes (Dolgin et al. 2007; Chaston et al. 2011). Third, a strategy of inbreeding and then high‐dose propagation is vulnerable to the spontaneous evolution of cheaters during laboratory culture (as diversity is restored by mutation). In contrast, a strategy of propagation with high relatedness infections will consistently select against cheaters and be robust to the invasion of less desirable genotypes over the long term.

## Data archiving statement

Data available from the Dryad Digital Repository: http://dx.doi.org/10.5061/dryad.c6829.

## Supporting information


**Figure S1**. Change in infectivity over passage experiment, infectivity being proportion of insects successfully invaded at high rate (500 IJs per dish).
**Figure S2**. The relationship between virulence (time to death ± SE) and nematode reproduction within and between assays at passage 6 and 12.
