# Supplementary material for: Limiting opportunities for cheating stabilizes virulence in insect parasitic nematodes
Source: Evol Appl. 2016 Jan 27;9(3):462–70. doi: 10.1111/eva.12348 (PMC4778107; doi:10.1111/eva.12348)
Supplement: Supplementary file 1 — Figure S1. Change in infectivity over passage experiment, infectivity being proportion of insects successfully invaded at high rate (500 IJs per dish). Figure S2. The relationship between virulence (time to death ± SE) and nematode reproduction within and between assays at passage 6 and 12. [file EVA-9-462-s001.pdf]

Figure S1. Change in infectivity over passage experiment, infectivity being proportion of insects successfully invaded at high rate (500 IJs per dish). Data are treatment means +SE based on normal approximation to the binomial. Open bar at PO indicates the baseline. *N* = 60 for baseline, and *N* = 80 for all other experiments.

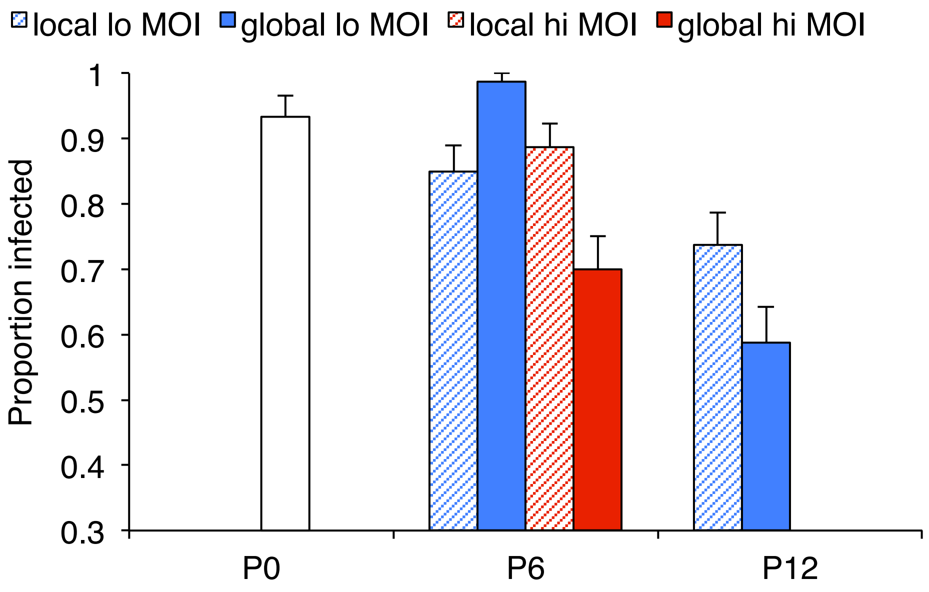

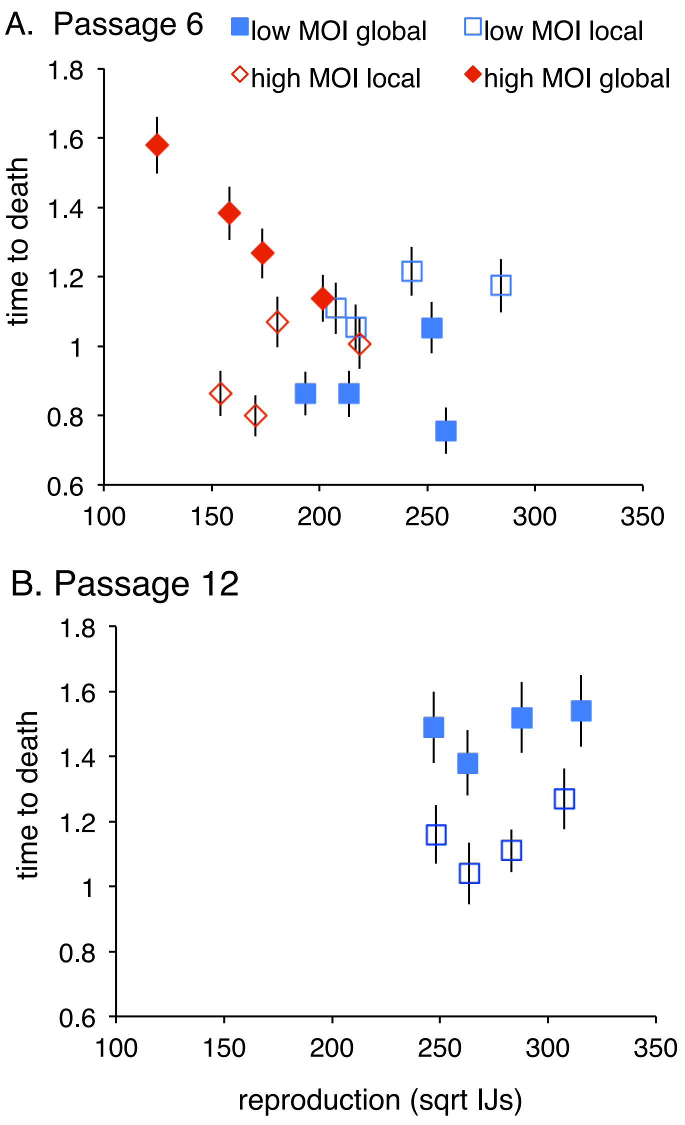

Figure S2. The relationship between virulence (time to death±SE) and nematode reproduction within and between assays at passage 6 and 12. **A.** At passage 6 there was no significant correlation between time to death (as estimated by survivorship models) and nematodes ($P = 0.2$) **B.** nor were there obvious correlations between these variables at passage 12.
